# Comparative Evaluation of the Effectiveness of Demineralized Freeze-Dried Bone Allografts With Titanium Platelet-Rich Fibrin and Autologous Dentin Graft With Titanium Platelet-Rich Fibrin for Alveolar Socket Preservation: Protocol for a Randomized Controlled Trial

**DOI:** 10.2196/70649

**Published:** 2025-12-10

**Authors:** Shivani Thakre, Pavan Bajaj

**Affiliations:** 1 Department of Periodontology Sharad Pawar Dental College Datta Meghe Institute of Medical Sciences Wardha, Maharashtra India

**Keywords:** demineralized freeze-dried bone allograft, DFDBA, autologous dentin graft, ADG, titanium platelet-rich fibrin, T-PRF, socket preservation, cone beam computed tomography, CBCT, histomorphometric analysis

## Abstract

**Background:**

Postextraction ridge resorption compromises the aesthetics and function of the subsequent implant therapy or prosthetic rehabilitation. Several techniques are employed to reduce ridge resorption, ranging from basic socket fillers such as blood clot preservation to more advanced techniques using barrier membranes and bone grafts such as autografts, allografts, xenografts, and synthetic materials.

**Objective:**

The aim of this study is to evaluate and compare the effectiveness of alveolar socket preservation by using demineralized freeze-dried bone allograft (DFDBA) with titanium platelet-rich fibrin (T-PRF) and autologous dentin graft (ADG) with T-PRF. This assessment will be performed through comprehensive clinical, radiographic, and histomorphometric analyses. The primary objective of this study is to histologically evaluate the new bone formation in the extraction sockets preserved using either DFDBA with T-PRF or ADG with T-PRF. The secondary objective is to evaluate the dimensional changes, including ridge width and height at these healing points, as measured by clinical and radiographic methods. We will evaluate and compare the clinical, radiographic, and histomorphometric outcomes of using DFDBA with T-PRF versus ADG with T-PRF for maintaining socket integrity after tooth extraction. We will also ascertain which grafting technique facilitates the production of new bone during the healing phase and preserves alveolar ridge dimensions.

**Methods:**

This randomized controlled trial will involve 16 patients aged 22-60 years requiring tooth extractions and subsequent implant placement. Participants will be randomly assigned to one of the 2 groups: (1) socket preservation using DFDBA in combination with T-PRF or (2) socket preservation using ADG with T-PRF. At baseline, all the clinical variables will be assessed using UNC-15 probe and cone beam computed tomography radiographs. Extraction will be done atraumatically with minimal flap reflection by using periotomes. Postextraction sockets will be preserved using DFDBA+T-PRF or ADG+T-PRF. At 4 months, clinical and radiographic evaluations will be done, and the implant will be placed using a 2-stage protocol. Histomorphometric analysis will be performed after receiving bone samples during implant placement. At 3 months after implant placement, the second-stage surgery will be done.

**Results:**

Participant enrollment commenced in March 2024, and the study is scheduled to conclude postassessments and analyses by the end of 2025. The results of this study are anticipated to be accessible in late 2025. This study is not funded, and the results are expected to be published by 2026.

**Conclusions:**

This study represents valuable insights into the clinical effectiveness of 2 biologically driven socket preservation techniques. We hypothesize that the use of ADG combined with T-PRF will show similar or more effective outcomes in alveolar socket preservation demonstrated by enhanced bone formation and better maintenance of socket dimensions compared to DFDBA combined with T-PRF without increased morbidity.

**Trial Registration:**

Clinical Trials Registry – India CTRI/2024/05/068192; https://ctri.nic.in/Clinicaltrials/pmaindet2.php?EncHid=MTI1NzIz&Enc=&userName=

**International Registered Report Identifier (IRRID):**

DERR1-10.2196/70649

## Introduction

Dental implants are the most widely used and reliable treatment method for individuals with fully or partially edentulous arches. The time frame for implant insertion following extraction is currently the main concern of implant therapy rather than osseointegration. Alveolar socket preservation following tooth extraction is one of the most important dental advanced skills, as numerous conditions such as traumatic extraction techniques, periodontal diseases, tumors, infections, or cysts can cause the loss of alveolar bone [1]. Alveolar ridge resorption is one of the physiological alterations that takes place following tooth extraction. After tooth extraction, alveolar ridge modeling peaks over the first 4-6 weeks, as is well known, while progressive atrophy may persist over time at a slower rate [2]. Ridge atrophy results from a substantial degree of physiologic remodeling that occurs in the alveolar socket following tooth extraction [3]. To minimize stress to the alveolus and accomplish minimal socket enlargement, atraumatic extraction must be performed. Treatment options for ridge atrophy include several forms of bone and/or soft tissue augmentation; nevertheless, these methods are known to have varying degrees of success and predictability. It is believed that socket grafting, which requires the use of grafting materials with or without barrier membranes, reduces the amount of dimensional shrinkage that occurs in the alveolar socket following tooth extraction.

For the best results, an ideal bone graft should have osteoconduction, osteoinduction, and osteoproliferation properties [4]. A range of surgical methods and biomaterials have been created to enable socket preservation. For this objective, several bone grafting methods, including synthetic and natural graft materials, have been tried. The most often used, clinically approved, or modified bone allograft material for periodontal repair is demineralized freeze-dried bone allografts (DFDBA). Bone morphogenetic proteins (BMPs) are linked to the osteoinductive capacity of DFDBA; this particular BMP combination includes BMPs 2, 4, and 7. DFDBA breaks down at a faster rate, which promotes the growth of new bone [5]. Its primary application is in the reconstruction of osseous deformities.

Growth factors such as platelet-derived growth factor are present intracellularly and essential for the healing of wounds. Utilizing platelet concentrates has several benefits. They are crucial to regeneration. Their application has been effectively linked to bone and connective tissue repair. Platelet concentrates promote bioactivity and demonstrate nonstimulative properties. Titanium platelet-rich fibrin (T-PRF) is one of the third-generation platelet concentrates [6]. This platelet concentrate is recognized to remove the potentially harmful effect of silica, which is found in glass vacuum containers used to prepare PRF. Platelet and leukocyte cell–enriched fibrin or T-PRF is made with titanium and resembles fibrin made with the traditional PRF process. Titanium is the most corrosion-resistant metal with the highest amount of toughness. Furthermore, titanium has an amazing osseointegration quality [6]. T-PRF presents a longer resorption time and a denser fibrin structure compared to PRF, making it a promising option for the sustained release of growth factors [7].

Histologically stated, hydroxyapatite makes up the majority of the inorganic structure that makes up human enamel and dentin, which is composed of 45% organic material. Type I collagen and BMP comprise most of the organic components. An autologous dentin graft (ADG) is a viable substitute for alveolar socket preservation due to its composition of organic and inorganic components as well as its ability to promote bone formation and support bone growth [4-6]. The osteoconductive characteristics of human dentin are attributed to 4 types of calcium phosphate: amorphous calcium phosphate, tricalcium phosphate, octacalcium phosphate, and hydroxyapatite. As hydroxyapatite in dentin is primarily found as calcium phosphate with a low crystal concentration, osteoclast activity can readily break it down. Approximately 90% of its 20% organic content is composed of type I collagen network; the remaining 10% is made up of noncollagenous proteins (such as phosphoprotein, sialoprotein, osteocalcin, and osteonectin, which help calcify bone), and 10% is made up of growth factors (like insulin-like growth factor and BMPs, which give teeth their osteoinductive properties) [8]. Owing to all these properties, it can be considered as potential graft material and has been used in the past for regenerative procedures.

Accurate assessments need the establishment of a consistent point for cone beam computed tomography (CBCT) linear measurements. CBCT is currently the most widely used 3D radiographic imaging technique in dentistry, offering several benefits over traditional CT scans and 2D imaging instruments. A more precise method involves using subtraction analysis of CBCT data [9]. The fundamental advantage of using CBCT in implant dentistry is its ability to provide detailed volumetric imaging data of the craniofacial region for preoperative and diagnostic planning.

CBCT radiographs have been reported to sometimes show certain discrepancies; hence, to eliminate those in the results, the outcomes will be confirmed by histomorphometric analysis. The bone sample for the histomorphometric analysis will be obtained using trephine bur during implant placement [10,11].

Hence, in this investigation, the aim is to clinically, radiographically, and histomorphometrically evaluate the effectiveness of DFDBA with T-PRF and that of ADG with T-PRF for socket preservation.

## Methods

### Study Design

This research is a clinical trial with randomized controlled prospective parallel arms to achieve a side-by-side comparison between ADG with T-PRF and a commercially available DFDBA bone graft with T-PRF.

### Ethical Considerations

This study was approved by the Institutional Ethics Committee of Datta Meghe Institute of Higher Education and Research, Wardha, Maharashtra, India (DMIHER(DU)/IEC/2024/226). All participants were informed about the study objectives, procedures, potential risks, and benefits prior to enrollment. Participation in this study was completely voluntary, and individuals were free to withdraw or opt out at any time without providing a reason and without any effect on their ongoing or future dental care at the institution. All data collected from participants were anonymized prior to analysis. Personal identifiers (name, address, phone number, and registration details) were removed and replaced by coded ID numbers. Access to the linking file was restricted to the principal investigator and stored on a password-protected institutional server to ensure confidentiality and data security. Participants did not receive any monetary compensation for taking part in the study. However, all clinical procedures performed as part of the research protocol were provided free of cost, ensuring no financial burden on participants. As no payment was made in foreign currency, no USD exchange rate was applicable. Written informed consent was obtained from all participants prior to their inclusion in the study, in accordance with the Declaration of Helsinki and institutional ethical guidelines.

### Study Population

Based on the necessity for a single tooth replacement via implant fixture, 16 healthy individuals without a history of any systemic disease will be enrolled in this research from the Department of Periodontics, Sharad Pawar Dental College, Sawangi (Meghe), Wardha. The participants will be informed about the study’s goal, methodology, and design prior to the study start. Written consent will be taken.

### Oversight and Monitoring

#### Composition of the Coordinating Center and Trial Steering Committee

The committee consists of the following: the head of the department of periodontics, postgraduate guide, research scientist, principal investigator, surgeon, statistician, data manager.

#### Composition of the Data Monitoring Committee

The data monitoring committee consisted of the research in-charge of the institute and research in-charge of the department.

#### Adverse Event Reporting and Harms

Data will be collected, assessed, and spontaneously reported during adverse events and other unintended effects of trial interventions or trial conduct.

#### Frequency and Plans for Auditing Trial Conduct

The project management group meet will review the trial conducted every month. The trial steering group and the independent data monitoring and ethics committee meet will review and conduct the trial period till the trial is complete.

### Eligibility Criteria and Recruitment

Following the clinical and radiographic assessments, 16 patients must precisely meet the criteria that are listed below.

#### Inclusion Criteria

The inclusion criteria are as follows.

1. Patients indicated for implant following tooth extraction for reasons such as residual roots, overretained primary teeth, nonrestorable carious lesions, internal and external resorption, fractured roots, and failed endodontic therapy
2. Patients with a complete mouth plaque score of less than 25%, indicating good oral hygiene
3. Patients with a natural tooth in opposition
4. Patients with adjacent teeth
5. Patients with a thick gingival biotype
6. Patients with a 4-mm bone apical to the root apex
7. Patients with bone quality of D-1, D-2, or D-3 type

#### Exclusion Criteria

The exclusion criteria are as follows.

1. Individuals with systemic conditions such as diabetes mellitus, osteoporosis, blood-related diseases, and titanium allergy that could compromise their overall health and impede the process of bone healing and repair
2. Individuals with a space between the maxilla and mandible
3. Individuals with parafunctional habits like clenching or bruxism
4. Individuals with a history of substance misuse, excessive smoking, or drinking
5. Individuals with D-4 bone quality
6. Individuals who have undergone chemotherapy and radiation
7. Individuals with temporomandibular joint disorders
8. Individuals with untreated oral health conditions
9. Pregnant and lactating mothers

### Recruitment

**Figure 1:**
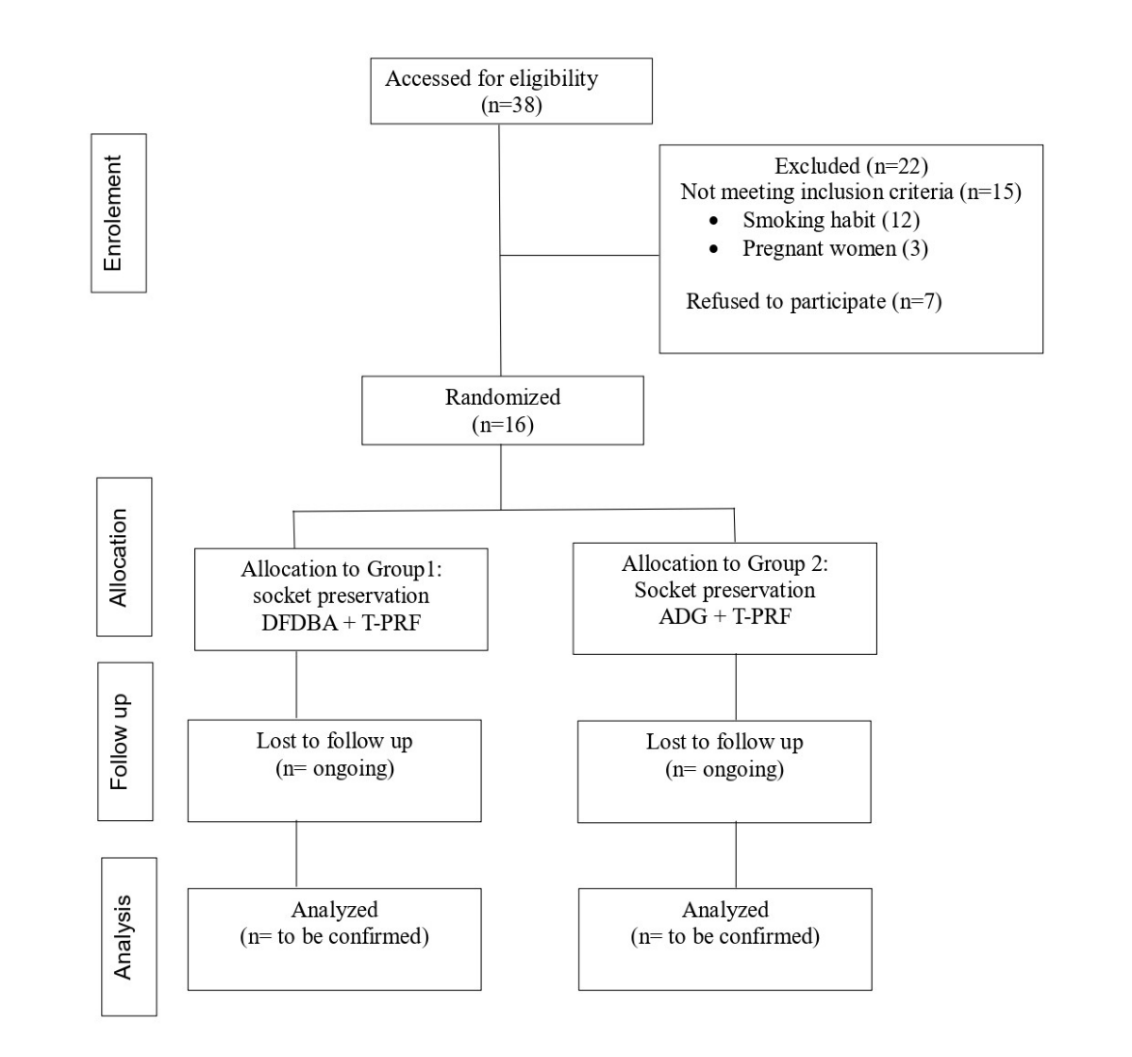
A simplified diagram that outlines the study protocol based on CONSORT (Consolidated Standards of Reporting Trials). ADG: autologous dentin graft; DFDBA: demineralized freeze-dried bone allograft; T-PRF: titanium platelet-rich fibrin.

Figure 1 presents a simplified diagram that outlines the study protocol based on the CONSORT (Consolidated Standards of Reporting Trials) guidelines (Multimedia Appendix 1).

### Primary Outcomes

Four months following the socket preservation procedure, the formation of new bone and changes in the alveolar socket dimensions in both groups will be assessed by radiological and histomorphometric analyses. Histomorphometric evaluations will be performed on bone core biopsies obtained at the time of implant placement at 4 months after the extraction. These samples will be processed using standard histological techniques, stained with hematoxylin and eosin, and assessed under light microscopy. Quantitative measurements such as the percentage of new bone formation, number of osteocytes, and trabecular patterns will be analyzed using Planmeca Romexis software (version 6.2.0; Planmeca Oy) to determine the measurement of bone dimensions, apico-coronal ridge height, and density in Hounsfield units.

### Secondary Outcomes

After 4 months of the surgical procedure, linear phenotypic dimensional changes in the thickness of soft tissues and the midfacial and midpalatal bones at baseline and at 4 months after healing at 2 locations will be evaluated.

### Sample Size Calculation

Sample size formula was calculated using mean (SD): 
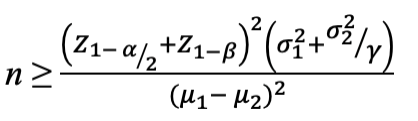
, where α=.01; β=.01; SD of newly formed bone with spontaneous healing (group 1), that is, group (δ_1_)=0.23; mean in group 1 (μ_1_)=0.72; SD of newly formed bone with ADG (group 2), that is, group (δ_2_)=0.24; mean in group 2 (μ_2_)=1.33; ratio (group 2/group 1)=1; and total samples required=8 per group. The calculation gives the result as sample required is 8 per group. The sample size was determined based on prior similar studies involving histomorphometric evaluation in socket preservation procedures. Based on the outcome variable on pocket depth with mean difference derived from previous literature [4], the sample size of 16 in total and 8 per group is adequate.

### Randomization and Masking

The study group consisting of socket preservation with ADG and T-PRF or the control group consisting of DFDBA and T-PRF will be randomly assigned. Every patient will be required to complete a comprehensive medical history form, give informed consent, and receive a thorough description of the study. A secure computer system will be used for central randomization in this procedure. A distinct study code will be given to each patient, and the operators and research evaluators will receive their data in a format that maintains patient confidentiality through coding. The radiologist and the statistician will be blinded to the treatment while analyzing the data. Blind quantitative histological analysis will be performed by a trained specialist.

### Clinical Procedure

#### Presurgical Phase

Patients will be clinically examined for periodontal diseases. Every patient will be required to complete a comprehensive medical history form, give informed consent, and receive a thorough description of the study. Following a comprehensive clinical examination and radiographical diagnosis, initial therapy will consist of guidance on maintaining good oral hygiene and a full mouth ultrasonic scaling procedure. Patients will receive plaque control instructions until their plaque score is less than 25%. Before any surgical procedure, a diagnostic cast will be made for every patient to ascertain the maxilla-mandibular relationship. Clinical photographs will be captured throughout the procedure. CBCT images will be obtained for every participant.

#### Preparation of T-PRF

Just prior to the surgery, by venipuncturing the antecubital vein, 10 mL of venous blood will be obtained under aseptic conditions and transferred to a new, sterile titanium tube. T-PRF is an autogenous blood concentrate of the third generation, characterized by a thick and durable fibrin meshwork that is triggered by a titanium tube [12]. Centrifugation is performed for 15 minutes at 3500 rpm. After transferring the resulting T-PRF clot onto a PRF box and separating it with sterile tweezers and scissors, the serum will be squeezed out of the clot to create a stable fibrin membrane [13]. A portion of the membrane is chopped up for use as graft material, and the remaining portion will be trimmed to fill the defect with membrane.

#### Surgical Procedure for the Test Group

**Figure 2:**
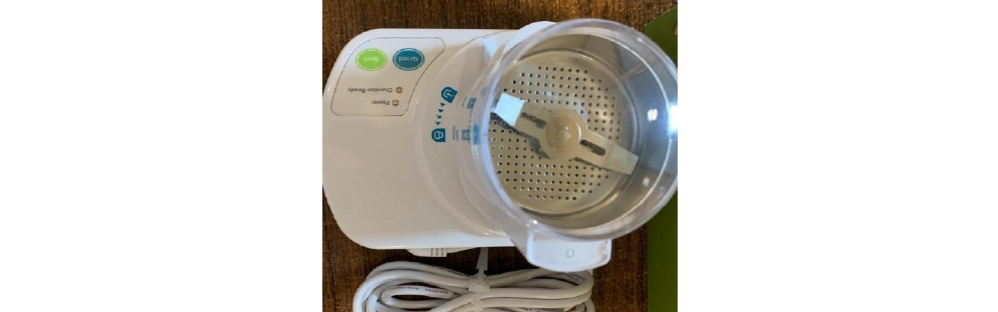
Dentin grinder.

Patients are instructed to gargle for 1 minute with 0.12% chlorhexidine gluconate. Local anesthesia will be administered. In order to minimize the surgical trauma during the flapless tooth extraction process, periotomes and forceps will be used. Using a number 15 blade, a crevicular incision will be created, removing periodontal ligament fibers on the distal and mesial sides. The teeth will be carefully luxated using elevators. Precautions will be taken to prevent trauma to the adjacent tissues. After tooth extraction, bone curettes will be used to completely debride the socket's granulation tissue. Initially, a high-speed handpiece will be used to remove any debris, artificial material, rot, and gutta-percha from the extracted roots, leaving just the clean tooth root. The roots that contain dentin will be crushed using a dentin grinder (Figure 2).

**Figure 3:**
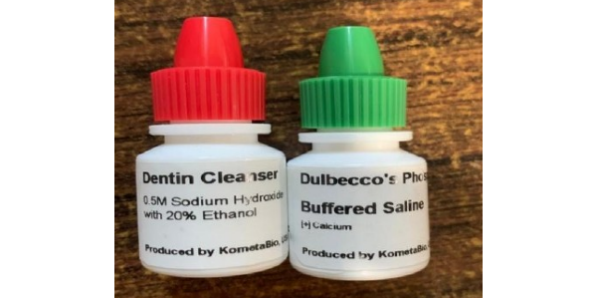
Dentin cleanser and buffered saline solutions.

The generated root dentin particle material will be treated through 3 steps. First, the dentin cleansing solution is poured into the dish containing the particles and allowed to sit at room temperature for 5 minutes. The solution is then allowed to dehydrate using a sterile gauze. Second, the particle is then totally covered with phosphate-buffered saline, which is then put into the plate (Figure 3).

**Figure 4:**
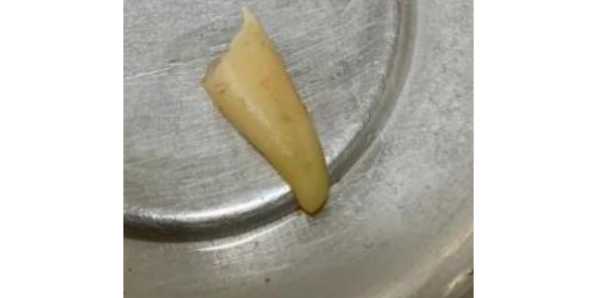
Extracted tooth sample.

The particle is mixed with fresh sterile gauze to dehydrate it by using a sterile device. With the phosphate-buffered saline solution, this step is repeated once more. To neutralize the pH levels, this step is crucial. At last, the ADG, whose particle size ranges from 300 to 1200 microns is prepared for prompt grafting (Figure 4).

**Figure 5:**
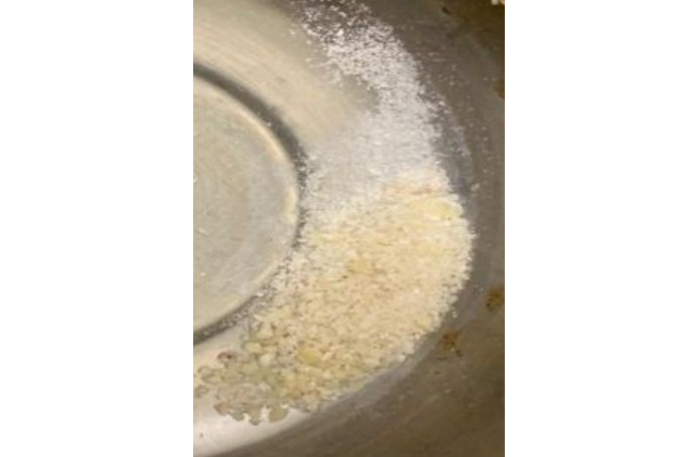
Prepared autologous dentin graft.

The extraction sockets will be filled with a mixture of T-PRF and ADG (Figure 5). After enclosing the resistant bone with a collagen plug, nonresorbable suturing material is used to sew the plug to the surrounding soft tissues.

#### Surgical Procedure for the Control Group

DFDBA, a completely resorbable alloplastic bone substitute, will be inserted into the extraction socket together with T-PRF following an atraumatic extraction. It is important to exercise caution when filling the socket after applying the graft since this could cause the exposed coronal particles to get sequestered or cause the graft mass to disperse as a whole. T-PRF is applied to compact the graft particles even more and accelerate in-situ healing.

#### Postsurgical Care

Participants will be given explicit instructions not to eat or drink anything hot on the surgical site nor should they rinse their mouths vigorously. All the patients will receive antibiotics (amoxicillin capsule 500 mg every 8 hours, metronidazole tablets 400 mg every 8 hours) and analgesics (Ibugesic tablets every 8 hours) after surgery for a period of at least 3 days. Two weeks following the surgical procedure, participants are recommended to gently brush the area surrounding the surgical site; nevertheless, regular tooth brushing should be continued for the remaining areas of the dentition starting on the day following the procedure. In addition, 2 weeks of twice-daily mouthwash containing 0.2% chlorhexidine is prescribed. Seven days following the procedure, the sutures will be removed.

#### Follow-Up

A complete examination will be conducted at the 4-month follow-up appointment [14]. Every clinical variable will be evaluated. A clinical and radiographic evaluation will be performed. Histomorphometric analysis will be performed on the bone obtained by trephine bur during implant placement.

### Statistical Analysis

Mean (SD) values will be found for each clinical periodontal parameter such as plaque index, papillary bleeding index, probing pocket depth, width of keratinized gingiva, clinical attachment level, and marginal bone level. Next, from baseline to 4 months, the mean data will be statistically analyzed. To equate baseline to 4-month findings for every patient, Student paired *t* test will be employed for intragroup data comparison, while the Student unpaired 2-sided *t* test will be utilized for intergroup comparison. If the *P* value exceeds .05, the observed difference will be considered nonsignificant; if it remains below .05, it will be considered significant.

## Results

This study received ethical approval in January 2024. Enrollment began from March 2024. The study commenced in August 2024 and is scheduled to conclude postassessments and analyses by the end of 2025. The results of the study are anticipated to be accessible in late 2025. Results will be shared using a variety of methods, including research reports, scholarly publications, and attendance at international conferences. A primary manuscript intended for submission to a prestigious, high-impact, peer-reviewed journal will elaborate on the findings related to the primary objective.

## Discussion

Socket preservation is a fundamental technique in dentistry, preserving alveolar bone structure after the extraction to facilitate optimal healing and support future dental restoration procedures. This study primarily focuses on histomorphometric analysis, which is considered a highly sensitive and accurate method for assessing new bone formation. Due to the precise and quantitative nature of histological outcomes, a smaller sample size can yield reliable and clinically meaningful data compared to studies relying solely on radiographic or clinical endpoints.

Hussain et al [4] demonstrated promising outcomes regarding the utilization of ADG in alveolar ridge preservation. Their study revealed superior dimensional stability in the study group compared to the control group, as evidenced by standardized CBCT scans. The study group's successful graft remodeling was further validated by histomorphometric analysis, demonstrating the potential of using ADG for effectively retaining the dimensional integrity of ridges. These findings highlight the need to utilize innovative biomaterials such as ADG to enhance clinical results and facilitate optimal wound healing.

Bhombe et al [5] investigated the use of PRF matrix and DFDBA as adjuvants during immediate implant placement procedures following tooth extraction. Their findings indicate that using PRFM and DFDBA in jumping gap distance as adjuvants resulted in significant coronal bone remodeling and a significant decrease in bone resorption.

In another clinical study by Elfana et al [15], histomorphometric findings demonstrated that the autogenous whole tooth and autogenous demineralized dentin graft groups exhibited higher proportions of newly formed bone (48.40%). The histology study confirmed that both types of transplants were compatible with the host tissues and showed no symptoms of inflammation.

Dwivedi et al [16] demonstrated that chair-side autogenous tooth grafts offer several advantages, including greater primary implant stability, faster recovery times, ease of preparation, reduced rates of bone resorption, and qualities that promote bone growth and socket augmentation.

ADG, which has both osteoconductive and osteoinductive qualities, appears to be a promising alternative for alveolar ridge preservation treatments, according to a systematic study by Sánchez-Labrador et al [17]. Positive outcomes in terms of volume preservation and histomorphometric data were noted; they were reinforced by the properties of materials, such as low complication rate and cost-effectiveness in comparison to alternative bone substitutes.

Thus, the purpose of this study is to assess and compare the effectiveness of DFDBA with T-PRF and ADG with T-PRF in socket preservation by performing clinical, radiological, and histomorphometric analyses. This study plan will be reliable to prove advantages pertaining to improvements in the maintenance of vertical and horizontal socket dimensions and phenotypic dimensions.

## Appendix

Multimedia Appendix 1 CONSORT 2010 checklist.

## Abbreviations

ADG: autologous dentin graft
BMP: bone morphogenetic protein
CBCT: cone beam computed tomography
CONSORT: Consolidated Standards of Reporting Trials
DFDBA: demineralized freeze-dried bone allograft
PRF: platelet-rich fibrin
T-PRF: titanium platelet-rich fibrin

## Data Availability

The datasets generated or analyzed during this study are available from the corresponding author on reasonable request.
